# Positioning of intraocular lens - capsular tension ring - capsular bag complex in eyes with compromised zonules: An endoscopic observation

**DOI:** 10.1016/j.ajoc.2026.102594

**Published:** 2026-05-04

**Authors:** Takahiro Shimowake, Takeshi Sugiura, Tetsuro Oshika

**Affiliations:** a Shimowake Eye Clinic, Ehime, Japan; b Sugiura Eye Clinic, Shizuoka, Japan; c Department of Ophthalmology, Faculty of Medicine, University of Tsukuba, Ibaraki, Japan

**Keywords:** Capsular tension ring, Zinn's zonule, Zonular integrity, Ciliary sulcus

## Abstract

**Purpose:**

To endoscopically assess the positioning of the intraocular lens (IOL)-capsular tension ring (CTR)- capsular bag complex in relation to the ciliary body in eyes with compromised zonular support.

**Degisn:**

Prospective, interventional case series.

**Methods:**

In four eyes with fragile zonules undergoing cataract surgery, a CTR was inserted during surgery, followed by IOL implantation into the capsular bag. An ophthalmic endoscope was introduced through a temporal corneal incision to visualize the positioning of the CTR-IOL-capsular bag complex relative to the ciliary body in the 180-degree quadrant opposite the corneal incision.

**Results:**

In two eyes, the equator of the CTR-IOL-capsular bag complex contacted the area slightly below the apex of the ciliary processes. In the other two cases, the equator of the complex was secured within the ciliary sulcus across the visualized region, effectively resolving phacodonesis which had been observed preoperatively and during capsulorhexis creation.

**Conclusions:**

The exploratory study provides the endoscopic evidence that the CTR-IOL-capsular bag complex can be fixed in the ciliary sulcus in some cases, potentially enhancing postoperative stability in eyes with compromised zonules.

## 1 Introduction

Capsular tension rings (CTRs) are designed to counteract zonular weakness during cataract surgery, thereby enhancing intraoperative safety and promoting postoperative intraocular lens (IOL) stability.^1^ Nevertheless, clinical outcomes regarding stability following CTR implantation demonstrate considerable variability.^2^ Some eyes exhibit persistent postoperative instability, and the IOL–CTR–capsular bag complex may ultimately dislocate into the vitreous cavity. Conversely, CTR-assisted surgery can effectively stabilize the lens in eyes exhibiting preoperative phacodonesis, eliminating even subtle pseudophacodonesis. This variability in outcomes is likely influenced by factors including the initial extent of zonular damage, the progressive nature of the zonulopathy, and other patient-specific variables.

In addition to these intrinsic factors, the intraoperative positioning and fixation status of the IOL–CTR–capsular bag complex, in relation to the ciliary body, are hypothesized to be critical determinants of long-term stability. However, no prior study has studied the fixation position of this complex, either during or after cataract surgery. To address this gap, we employed ophthalmic endoscopy intraoperatively to directly visualize the relative orientation and fixation of the CTR–IOL–capsular bag complex with respect to the ciliary body. Our study provides the first endoscopic evidence detailing the exact intraoperative seating and fixation of this complex, offering novel insights into mechanisms underlying sustained stability versus late dislocation.

## 2 Method

### 2.1 Study design and patients

This prospective interventional study included four eyes of three patients undergoing cataract surgery at the Sugiura Eye Clinic between December 2023 and June 2024. Patients’ background characteristics are shown in Table 1. All eyes demonstrated zonular fragility preoperatively or intraoperatively, and CTR-assisted cataract surgery was conducted.

**Table 1:** Patients’ background characteristics.

	Case 1		Case 2	Case 3
Age (years)	86		73	73
Gender	Male		Female	Male
Eye	L	R	R	R
Axial length (mm)	22.95	23.05	23.74	26.20
Angle-to-angle distance (mm)	11.19	10.89	11.82	11.65
Ocular comorbidities	Exfoliation, inadequate mydriasis	Exfoliation, inadequate mydriasis	Glaucoma, exfoliation, inadequate mydriasis, phacodonesis	Glaucoma, phacodonesis
Preoperative lens decentration and tilt on AS-OCT	-	-	-	-
Use of Malyugin ring	+	+	+	-
CTR inserting method	Manual	Spiral CTR injector	Spiral CTR injector	Spiral CTR injector
IOL brand	KS-AiN (STARR)	KS-AiN (STARR)	PN6A (KOWA)	PN6A (KOWA)
IOL total length (mm)	12.5	12.5	13.0	13.0
Position of CTR and haptics	Ciliary process	Ciliary process	Ciliary sulcus	Ciliary sulcus
Clinical outcomes at 6 months postoperatively				
Corrected visual acuity	20/20	20/20	20/15	20/15
Intraocular pressure	14	12	16	11
Complications	-	-	-	-

AS-OCT: anterior segment optical coherence tomography, CTR: capsular tension ring, IOL: intraocular lens.

Preoperatively, axial length was measured using optical biometry (AL4000, TOMEY Co., Ltd., Nagoya, Japan), and angle-to-angle distance was measured using anterior segment optical coherence tomography (AS-OCT, CASIA II, TOMEY Co., Ltd., Nagoya, Japan). Preoperative lens decentration and tilt were evaluated using AS-OCT.

### 2.2 Surgical technique

All procedures were performed through a temporal corneal incision. The timing and method of CTR insertion varied by case: in two eyes, the CTR was placed after phacoemulsification and irrigation/aspiration (IA); in the other two eyes, it was inserted before initiating phacoemulsification. In one eye, the CTR was inserted manually through a side port following phacoemulsification and IA, while in three eyes it was delivered using a Spiral CTR Injector (ASICO, Westmont, IL, USA).^3^

The same CTR model (130P, Hoya Surgical Optics, Tokyo, Japan) was used in all cases. This device has a 13.0 mm open diameter, an 11.0 mm compression diameter, and is composed of pigment-containing polymethyl methacrylate (PMMA) with a cross-sectional diameter of 0.18 mm.

In eyes with suboptimal mydriasis, a Malyugin ring (MST Co., Ltd., Redmond, USA) was used to achieve adequate pupillary dilation.

### 2.3 Endoscopic evaluation

Intraoperative endoscopy was performed immediately after CTR placement and again following IOL implantation. Imaging was conducted using an ophthalmic endoscope (VIT-20MNZ-HS, Machida Endoscope Co., Ltd., Tokyo, Japan) coupled with a light guide (RLED-300, Machida Endoscope Co., Ltd., Tokyo, Japan). The endoscope is designed for vitreoretinal surgery, with the following specifications: 20-gauge size, insertion diameter of 0.9 mm, a 75° field of view, and a minimum focal distance of 2 mm. The same manufacturer offers endoscopes in 23-gauge and 25-gauge sizes. However, we chose the 20-gauge endoscope because it provides a wider field of view compared to the 23- and 25-gauge models (65°). Furthermore, the smaller gauges suffer from reduced light intensity, resulting in darker images. The endoscope was introduced through the temporal corneal incision to visualize the positioning of the equator of the CTR-IOL-capsular bag complex relative to the ciliary body structures across a 180-degree arc opposite the site of corneal entry.

### 2.4 Ethics

The endoscope used in this study is engineered for vitreoretinal surgery; its application during cataract procedures therefore constituted an off-label use associated with additional invasiveness, prolonged operative time, and an increased number of procedural steps relative to standard practice. All patients were comprehensively informed of the off-label nature and potential risks, and written informed consent was obtained from every participant prior to enrollment. The study was conducted in accordance with the tenets of the Declaration of Helsinki and was approved by the Ethics Committee of the Sugiura Eye Clinic (approval number: 21000131).

## 3 Result

### 3.1 Cases 1

**Fig. 1:**
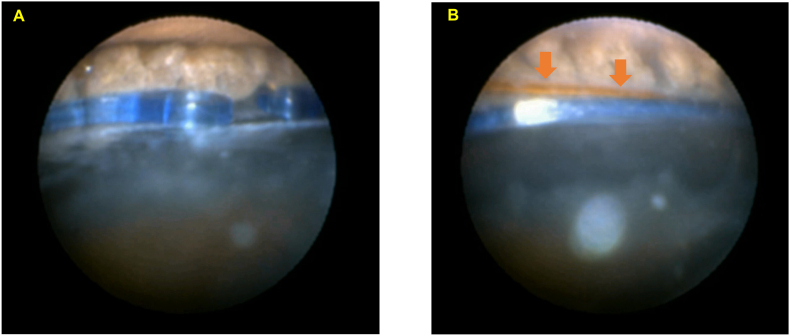
Endoscopic findings in the left eye of Case 1 A: Both ends of the CTR (dark blue) are positioned in contact with the apex of the ciliary processes at the 6 o'clock position. B: The IOL haptic (brown arrow) is observed just above the CTR at the 8 o'clock position. (For interpretation of the references to colour in this figure legend, the reader is referred to the Web version of this article.)

**Fig. 2:**
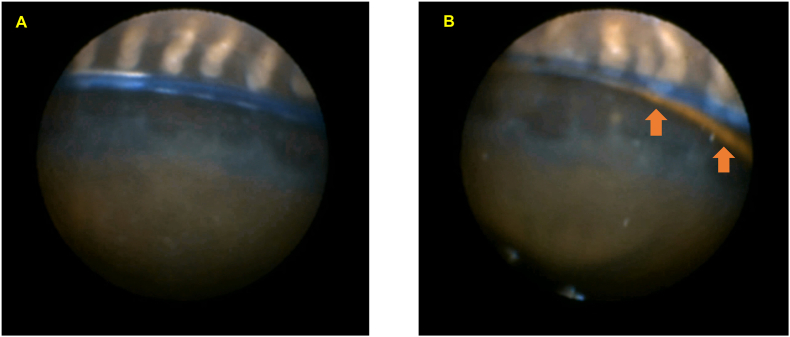
Endoscopic findings in the right eye of Case 1 A: The CTR is in contact with the apex of the ciliary process at the 3 o'clock position. B: Both the IOL haptic (brown arrow) and CTR (dark blue) are positioned just beneath the ciliary process apex at the 6 o'clock position. (For interpretation of the references to colour in this figure legend, the reader is referred to the Web version of this article.)

An 86-year-old male with exfoliation, zonular fragility, and poor pupillary dilation underwent bilateral cataract surgery. Preoperative AS-OCT revealed no lens decentration, tilt, or other morphological abnormalities. In the left eye, the CTR was manually inserted via a side port after IA. In the right eye, the CTR was inserted using a Spiral CTR Injector following IA. Intraoperative endoscopic examination revealed identical positioning in both eyes: the CTR and IOL haptics were located at or slightly below the apex of the ciliary processes in both eyes (Fig. 1, Fig. 2).

### 3.2 Case 2

**Fig. 3:**
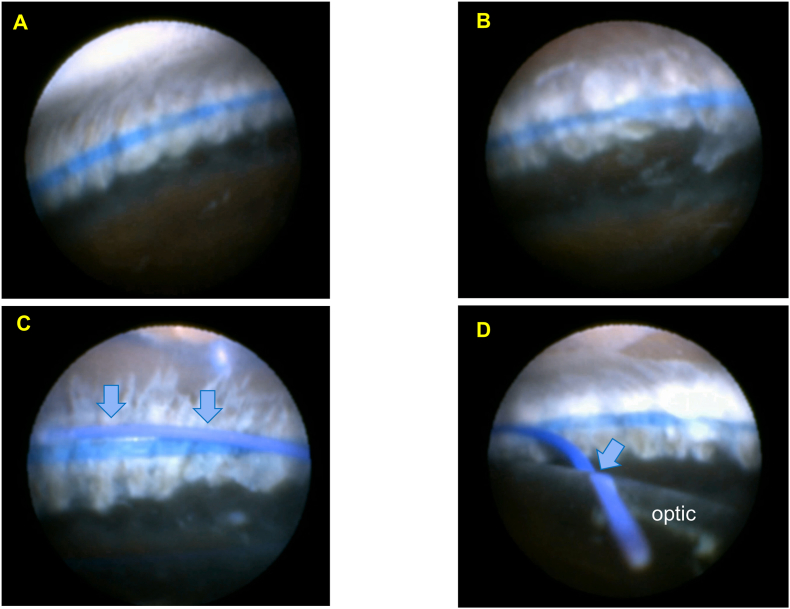
Endoscopic findings in Case 2 A: The CTR is secured on the anterior surface of the ciliary sulcus at 1-2 o'clock. B: The CTR is in contact with the apex of the ciliary processes at 4-5 o'clock. C: Following IOL implantation, the CTR (dark blue) and haptic (light blue arrow) are co-localized on the anterior surface of the ciliary sulcus at 1-2 o'clock. D: The CTR (dark blue) and haptic (light blue arrow) are fixed in the ciliary sulcus at 4-5 o'clock. (For interpretation of the references to colour in this figure legend, the reader is referred to the Web version of this article.)

**Fig. 4:**
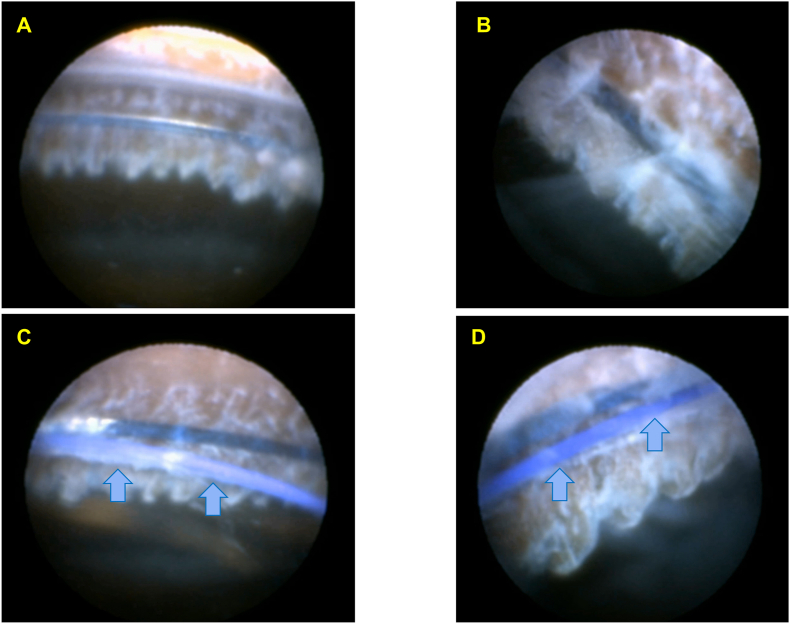
Endoscopic findings in Case 3 A: The CTR is positioned at the deepest part of the ciliary sulcus at 3 o'clock. B: The CTR is fixed at the deepest part of the ciliary sulcus at 6 o'clock. C: Following IOL implantation, the CTR (dark blue) and haptic (light blue arrow) are co-localized on the anterior surface of the ciliary sulcus at 3 o'clock. D: The CTR (dark blue) is secured at the deepest part of the ciliary sulcus while the haptic (light blue arrow) is located on the anterior surface of the ciliary sulcus at 12 o'clock. (For interpretation of the references to colour in this figure legend, the reader is referred to the Web version of this article.)

A 73-year-old female with exfoliation syndrome presented with severe zonular weakness, preoperative phacodonesis, and poor pupillary dilation necessitating Malyugin ring insertion. Lens decentration, tilt, and other morphological abnormalities were not detected on preoperative AS-OCT. The CTR was implanted using a Spiral CTR Injector prior to phacoemulsification. Endoscopic examination after IA revealed asymmetric CTR positioning: the device was secured in the ciliary sulcus above the ciliary processes at the 1-2 o'clock meridian (Fig. 3A) and at the apex of the ciliary processes at the 4-5 o'clock position (Fig. 3B). Following IOL implantation, the CTR and haptics co-localized in the ciliary sulcus at both the 1-3 o'clock (Fig. 3C) and 4-5 o'clock positions (Fig. 3D).

### 3.3 Case 3

A 73-year-old male with glaucoma and phacodonesis demonstrated marked zonular compromise with lens instability during hydrodissection. No lens decentration, tilt, or other morphological abnormalities were observed using preoperative AS-OCT. The CTR was inserted using a Spiral CTR Injector before initiating phacoemulsification. Endoscopic visualization after IA confirmed CTR fixation in the ciliary sulcus above the ciliary processes (Fig. 4A–B). Following IOL implantation, the haptics were positioned in the ciliary sulcus, co-localizing with the CTR (Fig. 4C–D).

At six months postoperatively, none of the 4 eyes from 3 patients demonstrated IOL decentration, tilt, dislocation, or other complications.

## 4 Discussion

In this study, we aimed to characterize the in vivo positioning of the CTR-IOL-capsular bag complex. Our findings indicate that the complex settles either at the apex of the ciliary process or within the ciliary sulcus, with its equator consistently contacting the ciliary body surface.

The standard size of CTRs commonly used in cataract surgery typically have an open diameter around 12.0 to 14.5 mm and a compressed diameter around 11.0 to 12.0 mm.^4^^,^^5^ The CTR used in our series measures 13.0 mm when open and approximately 11.0 mm when compressed. Prior UBM studies by Biermann et al.^6^ estimated the inter-ciliary sulcus distance at 12.51- 12.19 mm, and Sugiura et al.^7^ reported a 0.535 mm offset from the deepest sulcus to the ciliary process apex. From these data, the inter–apex distance of the ciliary processes can be approximated at 12.0–11.7 mm. In our cohort, angle-to-angle distance ranged from 10.89 to 11.65 mm (Table 1). Assuming the inter–ciliary sulcus distance scales similarly, these values support the plausibility that a CTR can engage within the ciliary sulcus.

The IOL diameters were 12.5 mm in Case 1 and 13.0 mm in Cases 2 and 3 (Table 1), allowing the haptics to potentially seat in the ciliary sulcus. In Case 1, the haptics ultimately rested below the ciliary processes, likely because a relatively larger CTR had already seated inferior to the processes and guided the haptics to that plane within the bag. In Case 2, although the CTR initially appeared to align at the ciliary process apex, both the CTR and haptics were found within the sulcus after IOL implantation, suggesting that outward haptic forces shepherded the CTR into the sulcus. Notably, Case 3 had the greatest axial length but not the largest angle-to-angle distance (Table 1), underscoring that any relationship between axial length, angle-to-angle distance, and final CTR-IOL fixation site will require larger samples to clarify.

The timing and technique of CTR insertion may influence its final position. When a Spiral CTR Injector is used prior to phacoemulsification, the ring tends to traverse between the anterior lens surface and the posterior face of the anterior capsule, favoring a location above the capsular equator and, consequently, more frequent entry into the ciliary sulcus. Conversely, insertion via a side-port often directs the ring obliquely downward, promoting fixation below the equator and decreasing the likelihood of sulcus engagement.

Variability in zonular integrity is difficult to quantify intraoperatively, but anatomic correlates offer a rationale. Using electron microscopy, Nishida et al.^8^ described three zonular layers—anterior, equatorial, and posterior—from their capsular insertions (anterior equatorial, equatorial, and posterior equatorial, respectively). The anterior fibers are thickest and presumed strongest, whereas posterior fibers are thinner and sparser, with inter-fiber gaps. We speculate that in Cases 2 and 3, rupture of the equatorial and posterior fibers with concurrent weakening of the anterior fibers permitted CTR and haptic fixation within the sulcus. By contrast, in Case 1, despite differing insertion methods, the CTR settled below the ciliary processes, likely because intact equatorial fibers exerted strong outward traction at the capsular equator, guiding and stabilizing the ring at that level. Clinically, the presence of phacodonesis, as in Case 3, may serve as a practical indicator of whether sulcus fixation of the CTR and haptics is achievable.

Whether CTR and haptics remain durably seated in the ciliary sulcus may depend on postoperative capsular contractile forces. If capsular contraction reduces the complex diameter below the inter–apex distance of the ciliary processes, displacement may occur. This consideration argues—pending further study—for larger-diameter CTRs (e.g., Morcher Type 13B, 14.5 mm) to enhance stability in select eyes.

A potential adverse event of firm, circumferential sulcus lodging of the CTR is impaired aqueous flow into the anterior chamber, precipitating malignant glaucoma. Bochmann et al.^9^ reported seven such cases, plausibly related to tight sulcus fixation. Reported management includes removal of the CTR–IOL complex with vitrectomy and secondary IOL fixation. If the pathophysiology is indeed mechanical blockage by the CTR–capsule complex, an alternative strategy may be to create openings in the anterior and posterior capsule outside the IOL optic using a vitreous cutter, potentially restoring flow without IOL explantation.

This study has several limitations. First, the sample size was small (n = 4 eyes). Because the protocol involved an invasive, off-label use of a 20-gauge endoscope designed for vitreoretinal surgery during cataract procedures, the institutional ethics committee required that the investigation be conducted with a limited number of cases. In addition, recruitment was constrained to patients with zonular weakness who were willing to consent to the study's aims, making a large-scale study impracticable. The high cost of single-use endoscopic probes was also a significant limiting factor. Based on the current findings, we plan to conduct further evaluations in a larger cohort. Second, the observational field was restricted to 180°. Achieving 360° visualization would necessitate creating an additional incision of 20-gauge or larger on the side opposite the main wound; however, in cases with zonular weakness that demand delicate, meticulous surgery, further increasing invasiveness was not approved by the ethics committee. Third, the postoperative follow-up period was limited; longer-term observation is required to adequately assess stability. Fourth, we were unable to detect differences in postoperative outcomes attributable to the fixation position of the CTR-IOL-capsular bag complex. Although clinically no IOL decentration, tilt, or dislocation occurred during the follow-up period, more sensitive methods for evaluating stability—such as quantitative assessment of subtle pseudophacodonesis—would enable more detailed analyses. Development and application of such methods remain important future challenges.

In conclusion, the preliminary findings of this exploratory study suggest that, in eyes with zonular weakness, the CTR–IOL–capsular bag complex can seat within the ciliary sulcus and may be associated with enhanced postoperative IOL stability. To translate this into consistent clinical benefit, future studies should rigorously define preoperative and intraoperative predictors of sulcus fixation (e.g., quantitative assessments of zonular integrity and capsular dynamics), and refine device sizing and insertion techniques to maximize stability while minimizing complications.

## CRediT authorship contribution statement

**Takahiro Shimowake:** Writing – review & editing, Investigation, Data curation, Conceptualization. **Takeshi Sugiura:** Writing – review & editing, Investigation, Conceptualization. **Tetsuro Oshika:** Writing – original draft, Supervision, Project administration, Formal analysis.

## Patient consent

Consent to publish this case report has been obtained from the patients in writing.

## Authorship

All authors attest that they meet the current ICMJE criteria for authorship.

## Declaration of generative AI and AI-assisted technologies in the manuscript preparation process

During the preparation of this work the authors used OpenAI GPT-5 in order to check and refine grammar and phrasing. After using this tool/service, the authors reviewed and edited the content as needed and take full responsibility for the content of the published article.

## Financial disclosures

Tetsuro Oshika has received compensation as a consultant from Alcon Laboratories, Inc., Johnson & Johnson Vision, Santen Pharmaceutical, HOYA Medical, Topcon Healthcare, and Logic and Design. He has received research funding from Alcon Laboratories, Inc., HOYA Medical, Johnson & Johnson Vision, KOWA, Otsuka Pharmaceutical, Santen Pharmaceutical, Senju Pharmaceutical, Tomey Co., Topcon Healthcare, Futaba, and Chugai Pharmaceutical. He has received lecture honorarium from Alcon Laboratories, Inc., Glaukos Japan, HOYA Medical, Johnson & Johnson Vision, KOWA, Santen Pharmaceutical, Senju Pharmaceutical, Tomey Co., Topcon Healthcare, Novartis, Inami, Logic & Design, Chugai Pharmaceutical, and Bayer. None of the other authors has any financial or proprietary interest in any material or method mentioned.

## Declaration of competing interest

The authors declare the following financial interests/personal relationships which may be considered as potential competing interests: Tetsuro Oshika has received compensation as a consultant from Alcon Laboratories, Inc., Johnson & Johnson Vision, Santen Pharmaceutical, HOYA Medical, Topcon Healthcare, and Logic and Design. He has received research funding from Alcon Laboratories, Inc., HOYA Medical, Johnson & Johnson Vision, KOWA, Otsuka Pharmaceutical, Santen Pharmaceutical, Senju Pharmaceutical, Tomey Co., Topcon Healthcare, Futaba, and Chugai Pharmaceutical. He has received lecture honorarium from Alcon Laboratories, Inc., Glaukos Japan, HOYA Medical, Johnson & Johnson Vision, KOWA, Santen Pharmaceutical, Senju Pharmaceutical, Tomey Co., Topcon Healthcare, Novartis, Inami, Logic & Design, Chugai Pharmaceutical, and Bayer. None of the other authors has any financial or proprietary interest in any material or method mentioned. If there are other authors, they declare that they have no known competing financial interests or personal relationships that could have appeared to influence the work reported in this paper.

## Data Availability

No data are available.
